# The REPRISE project: protocol for an evaluation of REProducibility and Replicability In Syntheses of Evidence

**DOI:** 10.1186/s13643-021-01670-0

**Published:** 2021-04-16

**Authors:** Matthew J. Page, David Moher, Fiona M. Fidler, Julian P. T. Higgins, Sue E. Brennan, Neal R. Haddaway, Daniel G. Hamilton, Raju Kanukula, Sathya Karunananthan, Lara J. Maxwell, Steve McDonald, Shinichi Nakagawa, David Nunan, Peter Tugwell, Vivian A. Welch, Joanne E. McKenzie

**Affiliations:** 1 School of Public Health and Preventive Medicine, Monash University, 553 St. Kilda Road, Melbourne, Victoria 3004 Australia; 2 Centre for Journalology, Clinical Epidemiology Program, Ottawa Hospital Research Institute, Ottawa, Canada; 3 School of Epidemiology and Public Health, Faculty of Medicine, University of Ottawa, Ottawa, Canada; 4 School of BioSciences, University of Melbourne, Melbourne, Australia; 5 School of Historical and Philosophical Studies, University of Melbourne, Melbourne, Australia; 6 Population Health Sciences, Bristol Medical School, University of Bristol, Bristol, UK; 7 Mercator Research Institute on Global Commons and Climate Change, Berlin, Germany; 8 African Centre for Evidence, University of Johannesburg, Johannesburg, South Africa; 9 Stockholm Environment Institute, Linnégatan 87D, Stockholm, Sweden; 10 The SEI Centre of the Collaboration for Environmental Evidence, Stockholm, Sweden; 11 Clinical Epidemiology Program, Ottawa Hospital Research Institute, Ottawa, Canada; 12 Faculty of Medicine, University of Ottawa, Ottawa, Canada; 13 Evolution & Ecology Research Centre and School of Biological, Earth and Environmental Sciences, University of New South Wales, Sydney, Australia; 14 Centre for Evidence-Based Medicine, Oxford University, Oxford, UK; 15 Department of Medicine, Faculty of Medicine, University of Ottawa, Ottawa, Canada; 16 Bruyère Research Institute, Ottawa, Canada

**Keywords:** Reproducibility of Results, Replication, Transparency, Systematic reviews, Meta-analysis, Methodology, Quality

## Abstract

**Background:**

Investigations of transparency, reproducibility and replicability in science have been directed largely at individual studies. It is just as critical to explore these issues in syntheses of studies, such as systematic reviews, given their influence on decision-making and future research. We aim to explore various aspects relating to the transparency, reproducibility and replicability of several components of systematic reviews with meta-analysis of the effects of health, social, behavioural and educational interventions.

**Methods:**

The REPRISE (REProducibility and Replicability In Syntheses of Evidence) project consists of four studies. We will evaluate the completeness of reporting and sharing of review data, analytic code and other materials in a random sample of 300 systematic reviews of interventions published in 2020 (Study 1). We will survey authors of systematic reviews to explore their views on sharing review data, analytic code and other materials and their understanding of and opinions about replication of systematic reviews (Study 2). We will then evaluate the extent of variation in results when we (a) independently reproduce meta-analyses using the same computational steps and analytic code (if available) as used in the original review (Study 3), and (b) crowdsource teams of systematic reviewers to independently replicate a subset of methods (searches for studies, selection of studies for inclusion, collection of outcome data, and synthesis of results) in a sample of the original reviews; 30 reviews will be replicated by 1 team each and 2 reviews will be replicated by 15 teams (Study 4).

**Discussion:**

The REPRISE project takes a systematic approach to determine how reliable systematic reviews of interventions are. We anticipate that results of the REPRISE project will inform strategies to improve the conduct and reporting of future systematic reviews.

**Supplementary Information:**

The online version contains supplementary material available at 10.1186/s13643-021-01670-0.

## Background

Many researchers have expressed alarm at the variation in results of systematic attempts to come up with the same answer to the same research question [1]. There have been frequent failures to obtain the same results when reanalysing the data collected in a study using the same computational steps and analytic code as the original study (this is usually referred to as a lack of ‘reproducibility’) [2–4]. Similarly, results have often differed when conducting a new study designed to address the same question(s) of a prior study (this is usually referred to as a lack of ‘replicability’) [5–7]. Concerns about reproducibility and replicability have led to efforts to enhance the ‘transparency’ of studies, by guiding authors to improve the reporting of methods and make the underlying materials, such as data and analytic code, publicly accessible [8, 9]. Investigations of transparency, reproducibility, and replicability in research have been directed largely at primary studies [2, 10–17]. However, such issues are also critical for systematic reviews—which attempt to locate and synthesise findings from all studies addressing a particular question—given their influence on decision-making and future research [18].

A reproduction or replication of an original systematic review might yield different results for various reasons. For example, authors of an original review might have made errors throughout the review process, by failing to include an eligible study, or entering study data incorrectly in a meta-analysis. Alternatively, different results might arise due to different judgements made by review teams about how best to identify studies, which studies to include, which data to collect and how to synthesise results [19–23]. Understanding the extent to which results of systematic reviews vary when analyses are reproduced or the entire review process is replicated, and the reasons why, can help establish how reliable and valid synthesis findings are likely to be in general. Such knowledge can also provide comparative evidence on the value of different systematic review methods. For example, replications designed to evaluate the impact of using automation tools or abbreviated methods (such as having only one author screen articles to speed up the process) could help reveal what risks the use of such methods entail, if any, when compared to traditional methods [24].

The few previous investigations of the transparency, reproducibility, and replicability of systematic reviews and meta-analyses differ in scope. Some have evaluated transparency of published reviews, documenting how often systematic reviewers report methods completely (i.e., in sufficient detail to allow others to repeat them), or share review data, analytic code, and other materials (e.g., [25–30]). Some investigators have re-extracted data from studies included in a sample of published systematic reviews and documented any inconsistencies with data included in the original meta-analyses and with the recalculated meta-analytic effect estimate (e.g. [20, 31–34]). There have also been some cases where two independent review teams were contracted to conduct systematic reviews addressing the same question concurrently, to see if consistent results were obtained (e.g. [35, 36]). These investigations provide some insight into the extent of transparency, reproducibility and replicability of systematic reviews, but have been narrow in scope, focusing only on one aspect of the review process, or restricting inclusion to one type of review (e.g. Cochrane reviews) or to reviews with one type of included study (e.g. randomised trials of drugs, or psychology experiments).

Available research on the transparency, reproducibility and replicability of systematic reviews leaves many questions unanswered. We do not know the extent to which completeness of reporting is associated with replicability of systematic reviews. It is unclear to what extent factors such as journal policies are associated with sharing of data, analytic code and other materials in systematic reviews, and what facilitators or barriers exist towards reviewers adopting them. We do not know what stages of the systematic review process, such as the search, selection, data collection or synthesis, are most prone to discrepancies between an original and replicated review. To address these gaps, we aim to explore various aspects relating to the transparency, reproducibility and replicability of several components of systematic reviews with meta-analysis of the effects of health, social, behavioural and educational interventions. Specifically, the objectives of the project are to evaluate in a sample of systematic reviews of interventions:

1. How frequently methods are reported completely, and how often review data, analytic code and other materials (e.g. list of all citations screened, data collection forms) are made publicly available;
2. Systematic reviewers’ views on sharing review data, analytic code and other materials and their understanding of and opinions about replication of reviews;
3. The extent of variation in results when independently reproducing meta-analyses using the same computational steps and analytic code (if available) as used in the original review; and
4. The extent of variation in results when replicating the search, selection, data collection and analysis processes of an original review.

## Methods

### Overview

**Fig. 1:**
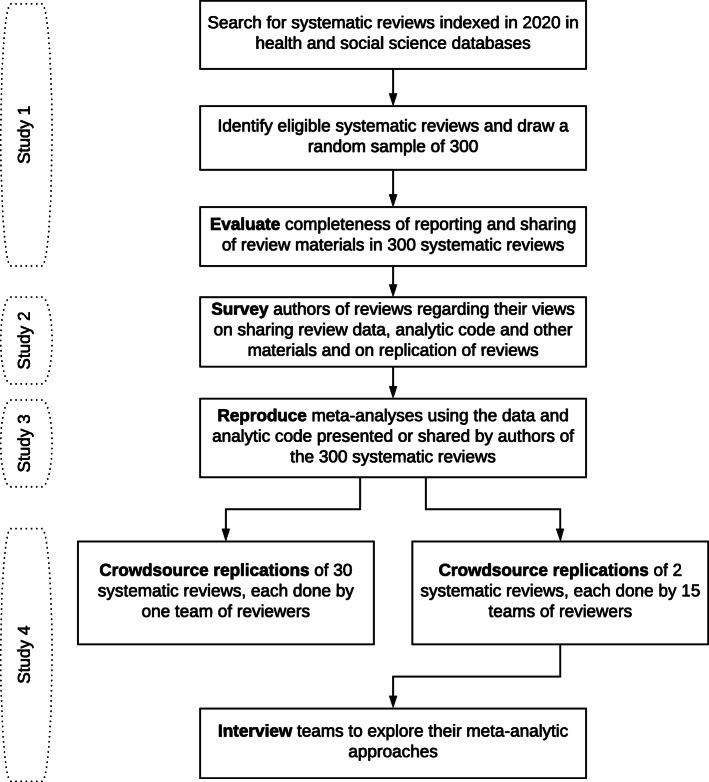
REPRISE project components

The REPRISE (REProducibility and Replicability In Syntheses of Evidence) project consists of a suite of studies to address our four objectives (Fig. 1). We will evaluate the completeness of reporting and sharing of review materials in a random sample of 300 systematic reviews with meta-analysis published in 2020 (Study 1). We will survey authors of systematic reviews to explore their views on sharing review data, analytic code and other materials and their understanding of and opinions about replication of systematic reviews (Study 2). We will then evaluate the extent of variation in results when we (a) independently reproduce meta-analyses using the same computational steps and analytic code (if available) as used in the original review (Study 3), and (b) crowdsource teams of systematic reviewers to independently replicate a subset of methods (searches for studies, selection of studies for inclusion, collection of outcome data, and synthesis of results) in a sample of the original reviews; 30 reviews will be replicated by 1 team each and 2 reviews will be replicated by 15 teams (Study 4).

We will focus on systematic reviews of the effects of health, social, behavioural and educational interventions. Eligible interventions will include any intervention designed to improve health (defined according to the World Health Organisation as “a state of complete physical, mental and social well-being and not merely the absence of disease or infirmity” [37]), promote social welfare and justice, change behaviour or improve educational outcomes. Examples of eligible interventions include inhaled corticosteroids to alleviate symptoms of asthma, provision of charity or welfare to alleviate social or economic problems, provision of regulations to improve safety in workplaces, use of bystander programs to prevent violence or harassment, or reduction of class size to enhance educational outcomes for high school students.

### Study 1: Evaluation of the transparency of systematic reviews with meta-analysis

The objective of this study is to evaluate the completeness of reporting and sharing of review data, analytic code and other materials (e.g. list of all citations screened, data collection forms) in systematic reviews with meta-analysis. We will do this by conducting a cross-sectional evaluation of systematic reviews published in 2020.

### Identification of systematic reviews

We will include a random sample of completed systematic reviews with meta-analysis in our evaluation. To be considered a “systematic review”, authors will need to have, at a minimum, clearly stated their review objective(s) or question(s), reported the source(s) (e.g. bibliographic databases) used to identify studies meeting the eligibility criteria, and reported conducting an assessment of the validity of the findings of the included studies, for example via an assessment of risk of bias or methodological quality. We will not exclude articles providing limited detail about the methods used (e.g. articles will be considered eligible if they provide only a list of the key words used in bibliographic databases rather than a line-by-line Boolean search strategy). Systematic reviews with meta-analysis meeting the following additional criteria will be eligible for inclusion in the study:

- Written in English, indexed in the most recent 1-month period closest to when the search for the present study is run, in one of the following bibliographic databases in the health and social sciences: PubMed, Education Collection via Proquest, Scopus via Elsevier and Social Sciences Citation Index and Science Citation Index Expanded via Web of Science;
- Includes randomised or non-randomised studies (or both) evaluating the effects of a health, social, behavioural or educational intervention on humans;
- Lists the references for all studies included in the review; and
- Presents at least one pairwise meta-analysis of aggregate data, including at least two studies, using any effect measure (e.g. mean difference, risk ratio).

Using search strategies created by an information specialist (SM), we will systematically search each database listed above for systematic reviews with meta-analysis meeting the eligibility criteria. For example, we will run the following search in PubMed: (meta-analysis[PT] OR meta-analysis[TI] OR systematic[sb]) AND 2020/11/02:2020/12/02[EDAT]. Search strategies for other databases are available in Additional file 1. We will download all records and remove duplicates using Endnote software, export the unique records to Microsoft Excel and randomly sort them using the RAND() function, then import the first 500 randomly sorted records into Covidence software [38] for screening. Two investigators will independently screen the titles and abstracts in batches of 500, retrieve any potentially eligible reports and independently assess each report against the eligibility criteria. This process will be repeated until we reach a target of 300 eligible systematic reviews. In the unlikely event that we do not reach our target of 300 reviews after screening all records yielded from the search, we will rerun the search to identify records published in the subsequent 1-month period and repeat the screening steps described above. We will include systematic reviews regardless of whether the question(s) they address are also addressed by another systematic review in the sample. Including 300 systematic reviews will allow us to estimate the percentage of reviews reporting a particular practice (e.g. reporting the full line-by-line search strategy) to within a maximum margin of error of 6% assuming a prevalence of 50% (i.e. 1.96*√(0.5*(1−0.5)/300)); for a prevalence of less (or greater) than 50%, the margin of error will be smaller.

### Data collection

Two investigators will independently and in duplicate collect information about each systematic review using a standardised data collection form. Any discrepancies in the data collected will be resolved via discussion or adjudication by another investigator. Prior to data collection, both investigators will pilot test the data collection form on a random sample of 10 systematic reviews within the set of 300, discuss any discrepancies and adjust the form where necessary. The form will include items capturing general characteristics of the review (e.g. journal it is published in, country of corresponding author, intervention under investigation, number of included studies) and items that characterise, for example, whether systematic reviewers:

- Reported the complete line-by-line search strategy used for at least one (or for each) electronic database searched;
- Reported the process for selecting studies, collecting data and assessing risk of bias/quality in the included studies;
- Presented the summary statistics, effect estimates and measures of precision (e.g. confidence intervals) for each study included in the first meta-analysis reported in the review; and
- Made data files and analytic code publicly available, and if so, specified how to access them.

We will evaluate the main systematic review report, any supplementary files provided on the journal server or in a public or institutional repository, or the review protocol if the authors specify that the relevant information is contained therein. The wording of items will be identical to that used in previous evaluations of this nature [25, 39] to allow investigation of improvements in transparency over time. We will record whether authors specified using a guideline such as the 2009 PRISMA statement [40] to guide reporting. We will also search the websites of the journals that published the included systematic reviews, and record whether or not they have a data or code sharing policy, or both, that is, a request that authors of all research articles or systematic reviews in particular share their data (or analytic code) when they submit an article [16]. If such a policy exists, we will extract it verbatim and classify it as “mandatory” (i.e. sharing data or analytic code, or both, is a condition of publication of systematic reviews by the journal) or “desirable” (i.e. authors are advised to share data or analytic code, but failing to do so will not preclude publication of systematic reviews).

### Data analysis

We will characterise indicators of transparency in the systematic reviews using descriptive statistics (e.g. frequency and percentage for categorical items and mean and standard deviation for continuous items). We will use risk ratios with 95% confidence intervals to examine differences in percentages of each indicator between reviews published in a journal that publishes evidence syntheses only (e.g. *Cochrane Database of Systematic Reviews*, *Campbell Systematic Reviews, JBI Evidence Synthesis*) versus published elsewhere; between the 2020 reviews of health interventions and a previously examined sample of 110 systematic reviews of health interventions indexed in MEDLINE in February 2014 [25, 39]; between reviews with self-reported use of a reporting guideline versus without; and between reviews published in journals with versus without *any* data or code sharing policy, and with versus without a *mandatory* data or code sharing policy.

### Study 2: Evaluation of systematic reviewers’ views on sharing review data, analytic code and other materials and on the replication of reviews

The objective of this study is to explore systematic reviewers’ views on sharing review data, analytic code and other materials (e.g. list of all citations screened, data collection forms) and their understanding of and opinions about replication of systematic reviews**.** We will gather systematic reviewers’ views via an online survey.

### Recruitment of systematic reviewers

Two investigators will independently screen the remaining titles and abstracts identified in Study 1 for inclusion in Study 2. We will invite all corresponding authors of systematic reviews of the effects of health, social, behavioural or educational interventions that meet the inclusion criteria for Study 1 to complete the survey, excluding authors of the randomly selected subsample of 300. The reason for excluding this subsample is to reduce author burden, since these authors will be contacted in Study 3. We will contact authors via email and send up to three reminders separated by 3 weeks in case of non-response. The survey will be administered via Qualtrics (Qualtrics, Provo, UT, USA).

### Survey content

The survey will capture demographic characteristics of the systematic reviewers (e.g. country of residence, career stage, number of systematic reviews conducted, areas of expertise) and their views on the open science movement. We will include questions asking authors to indicate the extent to which they agree that:

(i) Systematic reviewers should share review data, analytic code and other materials routinely;
(ii) Potential facilitators or barriers apply to the sharing of systematic review material, which we will draw from previous studies examining facilitators and barriers to adopting open science practices [41–47].

Responses will be collected via a 7-point Likert scale ranging from ‘strongly disagree’ to ‘strongly agree’. Authors will be given an opportunity to suggest other materials they believe systematic reviewers should share, and facilitators or barriers not listed in the survey. Finally, we will gauge authors’ understanding of and opinions about replication of systematic reviews, adapting the questions asked in previous studies evaluating researchers’ views on replication studies [48–50].

### Data analysis

We will analyse quantitative survey data by calculating the frequency and percentage for each response option for each question. We will use a deductive approach to coding of free-text responses to survey questions [51]. First, we will read each line of text and label it with a code that represents the meaning of the text; the initial codes will be informed by our prior work conducted to draft survey items. As each subsequent line of text is read, existing codes will be revised, where necessary, and new codes added, to ensure consistency of coding across responses. We will then organise codes into overarching themes. One investigator will perform the coding of text and categorisation of codes into themes, which will be verified by another investigator.

### Study 3: Evaluation of the reproducibility of meta-analyses

The objective of this study is to evaluate the reproducibility of meta-analyses. We will do this by evaluating the extent of variation in results when we independently reproduce meta-analyses using the same computational steps and analytic code (if available) as used in the original review.

### Sampling frame

We will include in this study all systematic reviews identified in Study 1 where the reviewers made available the data necessary to reproduce the first meta-analysis reported in the review, henceforth referred to as the “index meta-analysis”. If the systematic reviewers uploaded relevant data files (e.g. a Microsoft Excel or CSV spreadsheet, or a RevMan file containing all study effect estimates included in the index meta-analysis) or analytic code as a supplement to the paper, or reported a link to a publicly accessible repository (e.g. Open Science Framework, Dryad, figshare) or a website containing relevant files, we will download the files deposited. If no such files are referred to within the review report, or if the links to files are broken, we will invite the corresponding author of the review to share their systematic review data file(s) and analytic code for the purposes of reproduction, record what materials were shared, and request reasons for non-availability if materials were not shared. If no data file is made publicly accessible, one investigator will extract the study effect estimates included in the index meta-analysis from the relevant table or figure (e.g. forest plot) reported in the review. We will not extract or check for accuracy the data in reports of the included studies.

We anticipate obtaining data files (as a supplementary file to the paper, or from a public repository or the systematic reviewers) for at least 90 (30%) of the 300 reviews. In 2018, colleagues performing another study sought the aggregated data from 200 interrupted time series studies published from 2013 to 2017; they obtained the necessary data for 30% of studies from the study authors or as a supplementary file to the paper [52]. It is possible that the response rate will be higher in our study given the shorter time span between publication and us making the request (which will be less than 1-year post-publication).

### Reproduction of meta-analyses

Two investigators will independently carry out a reanalysis of the index meta-analysis of the review using the data available. For each meta-analysis, we will conduct the reanalysis according to the methods described in the published report of the review, regardless of how appropriate we consider the methods. If unable to conduct the reanalysis based on the data available and methods described, we will seek clarification from the systematic reviewers, and attempt to reanalyse the data based on the additional information provided. Each reanalysis will be conducted using the same statistical software package and version used by the original systematic reviewers, where possible. If systematic reviewers shared their analytic code, we will use it without modification. If no analytic code was shared, or if we are unable to access the software package and version used by the original systematic reviewers, we will write the code necessary to reanalyse the data ourselves, using the *metafor* package [53] in the open source statistical software R (R Development Core Team), based on the statistical methods described by the reviewers. We will record for each reanalysis the meta-analytic estimate of effect, its corresponding 95% confidence interval, and inferences about heterogeneity (Cochran’s *Q* and its *P* value, *I*^2^, tau^2^ and a prediction interval).

Two investigators will independently classify each index meta-analysis into one of three reproducibility categories:

(i) ‘Results fully reproducible’ (i.e. no difference [with allowance for trivial discrepancies such as those due to computational algorithms] is observed between the original and recalculated meta-analytic effect estimate, its 95% confidence interval and inferences about heterogeneity reported in the original review);
(ii) ‘Results not fully reproducible’ (i.e. a difference [even after allowance for trivial discrepancies] is observed between the original and recalculated meta-analytic effect estimate, its 95% confidence interval or inferences about heterogeneity reported in the original review, or;
(iii) ‘Results not able to be reproduced because of missing information’.

Two investigators will also independently specify whether they believe the observed difference between the original and recalculated summary estimate and its precision was meaningful, that is, would lead to a change in the interpretation of the results (classified as ‘difference meaningful’ or ‘difference not meaningful’). Any discrepancies in the classifications assigned by the two investigators will be resolved via discussion or adjudication by another investigator on the project. We will also record any difficulties in obtaining and using the author-supplied data or analytic code.

### Data analysis

We will calculate the frequency and percentage (with 95% confidence interval) of (i) systematic reviews for which a data file was made publicly accessible, (ii) systematic reviews for which analytic code was made publicly accessible, (iii) meta-analyses classified as having fully reproducible results *without* involvement of the original reviewer; (iv) meta-analyses classified as having fully reproducible results *with* involvement of the original reviewer, and; (v) differences between the original and recalculated summary estimate and its precision that were classified as meaningful. We will calculate agreement between the original and recalculated meta-analytic effects, displayed using Bland-Altman plots [54], and tabulate discordance between *P* values for the meta-analytic effects, by categorising the *P* values based on commonly used levels of statistical significance, namely *P* < 0.01; 0.01 ≤ *P* < 0.05; 0.05 ≤ *P* < 0.1; *P* ≥ 0.1. We will also classify any difficulties in obtaining and using the author-supplied data or analytic code into conceptually related themes to generate a list of common challenges experienced.

### Study 4: Evaluation of the replicability of systematic reviews

The objective of this study is to evaluate the replicability of systematic reviews. We will do this by evaluating the extent of variation in results when we crowdsource teams of systematic reviewers to independently replicate the searches for studies, selection of studies for inclusion in the review, collection of outcome data from studies and synthesis of results in a sample of the original reviews. By ‘crowdsource’ we mean recruiting a large group of individuals to complete the systematic review tasks [55–57].

We recognise that the terminology for ‘replication’ is not standardised within and across disciplines [7, 58]. In this study, we will adopt the non-procedural definitions of replication advocated by Nosek and Errington [5] and Machery [6]; that is, replicators will not need to follow every single step exactly as reported in the original systematic review, but they will be constrained by the original review question and must avoid making changes to the methods and concepts that might be reasonably judged to violate an attempt to answer that question.

### Sampling frame

We will use as our initial sampling frame the systematic reviews identified in Study 1 where the index (first reported) meta-analysis was reported completely. Specifically, meta-analyses in which the summary statistics (e.g. means and standard deviations per group) or an effect estimate (e.g. mean difference) and measure of precision (e.g. 95% confidence interval) were presented numerically for each study in a table or figure. We anticipate that such details will be available in at least 225 (75%) of the 300 systematic reviews, based on observations in previous evaluations of systematic reviews in medicine [25, 26] and psychology [30, 32]. For reasons of feasibility, we will then restrict the sampling frame to those reviews that included 5–10 studies in the index meta-analysis (which is likely to be the case in half of the reviews [39]), and in which searches of databases, registers or websites were carried out in English only. From this subset, we will draw a random sample of 32 reviews for replication by crowdsourced systematic reviewers (who we refer to as ‘replicators’).

### Crowdsourcing of reviewers

We will recruit replicators using approaches that have been used successfully in previous crowdsourced replication projects [10, 59–61]. We will send email invitations to our existing networks of systematic reviewer and methodologist collaborators (internal and external to the institutions we are affiliated with). These include investigators who contributed to the 2020 update of the PRISMA reporting guideline for systematic reviews [62]; members of the Society for Research Synthesis Methodology; and co-convenors of Cochrane Methods Groups, Campbell Methods Coordinating Group, and Joanna Briggs Institute (JBI) Methodology Groups, all of whom have extensive experience conducting systematic reviews and meta-analyses or developing methodology for evidence synthesis. Finally, we will advertise the project via our own social media channels and invite evidence synthesis organisations (e.g. Cochrane, Campbell Collaboration, JBI, Agency for Healthcare Research and Quality Evidence-based Practice Center Program, Collaboration for Environmental Evidence, 3ie, Global Evidence Synthesis Initiative, SPOR Evidence Alliance, Evidence Synthesis International) and journals that predominantly publish systematic reviews (e.g. *Cochrane Database of Systematic Reviews, The Campbell Library, BMC Systematic Reviews, JBI Evidence Synthesis*) to do so too. Via all these avenues, we will invite individuals to participate as a crowdsourced reviewer or recommend the opportunity to colleagues who may be suitable. As an incentive for participation, all replicators will be invited to contribute as authors on the main paper resulting from this study.

To be eligible to participate, replicators will need to have had experience with running a systematic search in a bibliographic database, collecting outcome data from studies or undertaking meta-analysis in at least one systematic review of any intervention within the previous 3 years. To confirm that researchers unknown to us have the necessary skills to participate, we will ask each to describe any relevant publications and training in systematic review methods, and to specify what tasks they contributed to in previous reviews. Three members of the research team will preside over an accreditation committee to ensure all replicators meet a similar standard. We aim to recruit at least 60 replicators who will form 30 teams, with each team including at least 2 members.

### Data collection

One of the REPRISE investigators will assemble all the information and files necessary for the replication of the 32 systematic reviews. This will involve extracting from each systematic review report (or review protocol or supplementary files, where necessary):

- The inclusion and exclusion criteria for the review, such as eligible participants, interventions, outcomes, study designs, languages of publication;
- Full details of the search methods, including the line-by-line search strategies for each database consulted, and dates when databases were last searched;
- Results of each study included in the index meta-analysis (i.e. summary statistics and effect estimate with a measure of precision, such as a confidence interval or standard error), and;
- Results of the meta-analysis (i.e. meta-analytic effect estimate with a measure of precision and inferences about heterogeneity).

We will prepare forms for replicators to record all screening decisions, data collected and synthesis results generated in the replication attempts, which they will be permitted to modify however they see fit. We will share all instructions and PDF copies of full-text reports with replicators via the Open Science Framework repository.

We will also invite the authors of the original reviews to provide us with a file containing all their screening decisions, if not already made publicly accessible. Examples of such files include a Microsoft Excel or Endnote file listing all titles and abstracts screened, and a list of citations for all full-text reports retrieved, and what the decision about eligibility was for each.

### Replication methods

Of the 32 reviews included, 30 will be replicated by one team each, who will be asked to address the question addressed by the index meta-analysis in the original review and provided detailed information (where available) about the methods used in the original review. We will strive to match the topic of the review to the expertise of the replicators. The remaining two reviews will be replicated by 15 teams, who will be asked to address the question addressed by the index meta-analysis in the original review but provided minimal information about the methods used in the original review. The purpose of the latter replication is to explore how different teams approach the synthesis of the same set of studies (using a “many analysts” approach [20, 60, 63]). For the two reviews replicated by 15 teams, we will strive to select reviews with multiplicity of results in the included studies, diversity in the study characteristics, and diversity in the risk of bias in the included studies and extent of missing evidence (e.g. unpublished studies), and which match the content expertise of the majority of replicators. Each team will conduct two replications (one of each type). We will ensure that none of the replicators are involved in the replication of an original review that they co-authored, of an original review that addresses the same or a similar question as another systematic review they have conducted, or of an original review which includes a study that they conducted. Replicators will be asked to sign an electronic contract confirming that they will not access the original review assigned to them (to prevent it from influencing their replication attempt).

### Replications by one team each

For the 30 systematic reviews replicated by one team each, replicators will initially undertake three tasks. First, one team member will re-run the search strategies for all bibliographic databases searched in the original review that they (or the REPRISE investigators) have an institutional subscription to; searches of other sources, such as trials registers and websites, will not be replicated. Searches will be run using the exact same search string and date limits as reported in the original review. Where possible, searches will be run so as not to retrieve records that were published within the date limits but indexed in the database *after* the search was originally run. If full search strategies are not reported, replicators will be permitted to request the strategies from the authors of the original systematic review (with the contact made via one of the REPRISE investigators). If authors of the original review reported or provided only the search terms used, not the full line-by-line search strategy, replicators will need to attempt to reconstruct a search strategy from the terms provided (and will be permitted to seek assistance from an information specialist if they do not have one on their team). If authors of the original review reported or provided a search strategy for one database only, replicators will need to attempt to reconstruct search strategies for the other databases by adapting the one provided. Replicators will document which original search strategies they attempted to rerun, which search strategies they needed to reconstruct or adapt based on the partial information available, any errors detected when rerunning each original search strategy, the number of citations that each database yielded, the total number of citations yielded across all databases consulted, and the number of unique citations after duplicates were removed.

Second, two team members will screen independently a random sample of a maximum of 100 titles and abstracts yielded from the searches against the inclusion criteria reported in the original review, and record their screening decisions (‘include’, ‘exclude’, or ‘unsure’). We have restricted this step to 100 records to minimise burden on replicators. Any discrepancies between the team members will be resolved via discussion or adjudication by another team member (or, if the team comprises two members only, by one of the REPRISE investigators who will be unaware of the studies included in the original review).

Third, we will retrieve and send to team members full-text reports of all the studies included in the original systematic review (not just those included in the index meta-analysis), and full-text reports of studies excluded from the original review (if cited, listed in a ‘table of excluded studies’, listed in a supplementary file or shared by the original authors on request). We will cap the number of excluded full-text reports to screen at 50, drawing a random sample if more than 50 were listed by the original reviewers. If information about full-text reports that were excluded is not available, we will send only full-text reports of the included studies to team members. Two team members will screen independently all study reports written in English that were assigned to them against the inclusion criteria reported in the original review, and record screening decisions (‘include’, ‘exclude’, or ‘unsure’). Any discrepancies between the team members will be resolved via discussion or adjudication by another team member (or, if the team comprises two members only, by one of the REPRISE investigators who will be unaware of the studies included in the original review).

For these three tasks, we will only provide teams with the background, rationale, objectives, search strategies, eligibility criteria and the screening form/checklist from the original review (where available), so that their screening decisions are not influenced by knowledge of which studies were included in the original review. Replicators will be permitted to contact the authors of the original systematic review for clarification if any of the study eligibility criteria were unclear to them (with the contact made via one of the REPRISE investigators). If replicators felt unable to re-run or reconstruct any search strategies because the methods reported were incomplete, or if the reported search strategies did not rerun properly, replicators will note this and avoid the second step (screening of titles and abstracts) given no citations will be available to screen.

Next, we will provide teams with reports of all studies included in the index meta-analysis and information about what to collect from each study. Such information will include the outcome domain (e.g. social isolation), experimental and comparator intervention (e.g. visiting by a volunteer versus no visiting), meta-analytic effect measure (e.g. mean difference) and any decision rules reported by the original systematic reviewers regarding which results to select from studies if multiple results were available (e.g. which measurement scale, time point or analysis sample was selected). Two replicators in the team will collect independently the outcome data from each study report that they deem most compatible with the index meta-analysis and the methods stipulated by the original reviewers. Any discrepancies in the data collected by team members will be resolved via discussion or adjudication by another team member or one of the REPRISE investigators. Where necessary, replicators will calculate a study effect estimate and its 95% confidence interval based on the summary statistics collected.

Using the data collected, replicators will then calculate a meta-analytic effect estimate, its 95% confidence interval and *P* value, and inferences about heterogeneity, according to the meta-analysis methods described in the report, using the statistical package they are most familiar with (e.g. R, Stata, RevMan, Comprehensive Meta-Analysis). Replicators will be permitted to contact the authors of the original systematic review to clarify missing or unclear details about how they did their analysis. Replicators will also be permitted to contact the authors of studies included in the index meta-analysis when essential data in the study reports were missing or ambiguous, regardless of whether the authors of the original systematic review did so. In both scenarios, one of the REPRISE investigators will make the contact. We will provide fields in the data collection form for replicators to record any challenges they experienced with recalculating study or meta-analytic results.

Once they have completed the replication attempt, we will provide teams with a table or figure (e.g. forest plot) indicating the study results included in the original meta-analysis. Each replicator will independently record, for each study included in the meta-analysis, whether the results they extracted from the study report (or via contacting the study authors) matched the results included in the meta-analysis. Also, one REPRISE investigator and the team who attempted to replicate the review will independently classify the meta-analysis into one of three replication categories:

(i) ‘Results fully replicable’ (i.e. no difference [with allowance for trivial discrepancies such as those due to computational algorithms] is observed between the original and recalculated meta-analytic effect estimate, its 95% confidence interval and inferences about heterogeneity reported in the original review);
(ii) ‘Results not fully replicable’ (i.e. a difference [even after allowance for trivial discrepancies] is observed between the original and recalculated meta-analytic effect estimate, its 95% confidence interval or inferences about heterogeneity reported in the original review, or;
(iii) ‘Results not able to be replicated because of missing information’.

A REPRISE investigator and the team will also independently specify whether they believe the observed difference between the original and recalculated summary estimate and its precision was meaningful, that is, would lead to a change in the interpretation of the results (classified as ‘difference meaningful’ or ‘difference not meaningful’). Any discrepancies in the classifications assigned by the REPRISE investigator and team will be resolved via discussion or adjudication by another REPRISE investigator.

### Replications by 15 teams

For the two systematic reviews replicated by 15 teams, we will provide teams with reports of all studies included in the review (not just those included in the index meta-analysis). For this review, we will instruct teams to attempt a synthesis of a specific outcome domain (e.g. delinquent behaviour) using the data available in the study reports; however, we will not tell them which studies or results were included in the original meta-analysis, or any decision rules the original systematic reviewers used to select results from studies when multiple were available. Each team will decide for themselves which data to select from the study reports (e.g. which time point or scale to select if multiple were available for the outcome domain), whether to synthesise the data, which studies and data to include in the synthesis, and how to synthesise the data (e.g. which meta-analysis model to use to synthesise results). Teams will be permitted, but not expected, to assess the risk of bias in the studies using any tool they deem suitable, to help inform which studies to include in the synthesis. Teams will then calculate study and synthesised effects using the statistical package they are most familiar with. Teams will need to submit to the REPRISE investigators a report specifying the analysis methods they used and the results they found, which we will de-identify to prevent identification of team members. Teams will not be permitted to publicise or discuss their methods and results with others until advised by the REPRISE investigators to ensure independent analyses across teams.

### Interview methods

Once teams complete both replications, investigators will conduct semi-structured interviews (of approximately 30 min duration) with replicators to discuss the analytical steps they took and to understand the decision-making processes used when synthesising the data. The interviews will be conducted by two investigators, one who will lead the discussion and the other who will listen and consider questions that may need to be asked for clarification or further exploration. We will focus particularly on decisions or steps for which replicators found there was insufficient (or no) operational detail to decide what to do. We will also gauge their views on the appropriateness of the methods used by the original reviewers, alternative statistical synthesis approaches that they could have undertaken (particularly those conducted by other teams), and any challenges experienced with conducting both replications. We will analyse the data after each interview and adjust our questioning in subsequent interviews where necessary, to explore important leads that we uncover. We will conduct the interviews using videoconferencing software, which we will audio record and transcribe verbatim.

### Data analysis

For the 30 systematic reviews replicated by one team each, we will assess agreement between the original and replicated review in the number of citations yielded from each database, in total and once duplicates were removed, by calculating the weighted Kappa statistic and percentage agreement (both metrics will be presented with 95% confidence intervals). We will use the same metrics to assess agreement between the original and replicated review in screening decisions (where available) for the subset of titles and abstracts and full-text reports screened by replicators. We will interpret Kappa values as poor (≤ 0.00), slight (0.01–0.20), fair (0.21–0.40), moderate (0.41–0.60), substantial (0.61–0.80) or almost perfect (0.81–1.00) [64]. We will calculate the frequency and percentage (with 95% confidence intervals) of (i) original search strategies that the replicators attempted to rerun, (ii) search strategies replicators needed to reconstruct or adapt based on the partial information available; (iii) original search strategies with errors detected when rerun; (iv) studies where the effect estimate and its precision collected or calculated (from summary statistics) matched the effect estimate and its precision included in the meta-analysis, (v) meta-analyses in each of the three replication categories and (vi) differences between the original and recalculated summary estimate and its precision that were classified as meaningful. We will calculate agreement between the original and replicated meta-analytic effects, displayed using Bland-Altman plots [54], tabulate discordance between *P* values for the meta-analytic effects, by categorising the *P* values based on commonly used levels of statistical significance, namely *P* < 0.01; 0.01 ≤ *P* < 0.05; 0.05 ≤ *P* < 0.1; *P* ≥ 0.1.

For each of the two systematic reviews that were replicated by 15 teams, we will visually display the meta-analytic effect estimate and 95% confidence interval generated by each team that chose to meta-analyse the data, and calculate the median, interquartile range and range of each value across the teams. We will also present the findings of teams who used an alternative statistical synthesis method or who chose not to use any statistical synthesis method (e.g. because they believed the studies were too clinically diverse to combine).

For the interview data, we will adopt the same approach used to analyse free-text responses to survey questions in Study 2. That is, one investigator will code the transcripts deductively, and codes and themes generated will be verified by another. We will also classify any difficulties in replicating any of the review methods or recalculating results into conceptually related themes to generate a list of common challenges experienced.

## Discussion

The REPRISE project takes a systematic approach to determine how reliable the findings of contemporary systematic reviews of interventions are. To our knowledge, our project will be the first to explore systematic reviewers’ views on sharing review data, analytic code and other materials, and the extent to which the data and analytic code shared by reviewers is reusable and their findings reproducible. The project will also be the first to evaluate replicability of multiple stages of the systematic review process (e.g. search and selection process, collection of data and statistical synthesis) and therefore enables us to determine which stages are most prone to discrepancies between an original and replicated review. The “many analysts” approach to evaluate inter-observer agreement in synthesis results has been explored only once previously [20], so our project will provide a current estimate of the extent of inter-observer agreement. To allow others to verify our findings, we will make all materials, de-identified data collected and analytic code used publicly accessible on the Open Science Framework repository at the completion of the project.

There are a couple of limitations of our planned approach. Compared to the studies included in some previous systematic replication attempts, which can be conducted quickly and inexpensively (e.g. [10–12]), a full systematic review can take anywhere between 6 months and 3 years to complete, depending on the volume of literature to screen and synthesise [65, 66]. Therefore, for reasons of feasibility, we plan to replicate only a subset of the methods used in systematic reviews rather than carry out full replications. Also, some of our eligibility criteria, such as the decision to limit to meta-analyses of 5–10 studies, and our focus on one meta-analysis in each review (the first reported one), mean that we will examine a subset of published systematic reviews. Therefore, our estimates of reproducibility and replicability may not generalise to all meta-analyses.

The REPRISE project should inform strategies to improve the conduct and reporting of future systematic reviews. With the production of systematic reviews and meta-analyses increasing exponentially [39, 67] and expanding into many other areas of science [68, 69], investment in strategies to improve their conduct and reporting could benefit the many different stakeholders who rely on synthesised evidence for decision-making. We anticipate that results of the REPRISE project will also draw attention to the potential value of replicating systematic reviews, which to date has been under-recognised by researchers, funders, journals and other stakeholders, and inform updates to guidance on when and how to replicate systematic reviews [18, 70].

## Supplementary Information

**Additional file 1:.** Search strategies

## Data Availability

No data are associated with the article.
