# Supplementary material for: The REPRISE project: protocol for an evaluation of REProducibility and Replicability In Syntheses of Evidence
Source: Syst Rev. 2021 Apr 16;10:112. doi: 10.1186/s13643-021-01670-0 (PMC8052676; doi:10.1186/s13643-021-01670-0)
Supplement: Supplementary file 1 — Additional file 1:. Search strategies [file 13643_2021_1670_MOESM1_ESM.pdf]

Supplementary Material to:

The REPRISE project: protocol for an evaluation of REProducibility and Replicability In Syntheses of Evidence

Matthew J Page*, David Moher, Fiona M Fidler, Julian PT Higgins, Sue E Brennan, Neal R Haddaway, Daniel G Hamilton, Raju Kanukula, Sathya Karunananthan, Lara J Maxwell, Steve McDonald, Shinichi Nakagawa, David Nunan, Peter Tugwell, Vivian A Welch, Joanne E McKenzie

***Correspondence to**: Dr. Matthew Page, School of Public Health and Preventive Medicine, Monash University, 553 St Kilda Road, Melbourne, Victoria, 3004, Australia. Email address: matthew.page@monash.edu

Supplement 1: Search strategies

Searches run Thursday 3 December 2020

PubMed
(meta-analysis[PT] OR meta-analysis[TI] OR systematic[sb]) AND 2020/11/02:2020/12/02[EDAT]

Science Citation Index Expanded (SCI-EXPANDED) and Social Sciences Citation Index (SSCI) via Web of Science
(TI=meta-analysis OR AB=meta-analysis OR TS=meta-analysis OR TI="systematic review" OR AB="systematic review" OR TS="systematic review") AND **LANGUAGE**: (English) AND **DOCUMENT TYPES**: (Article OR Review)
Indexes=SCI-EXPANDED, SSCI; Timespan=Last 4 weeks

Scopus via Elsevier
TITLE("meta-analysis" OR "systematic review") AND ORIG-LOAD-DATE > 1604275200 AND ORIG-LOAD-DATE < 1606867200 AND (LIMIT-TO(DOCTYPE, "ar") OR LIMIT-TO(DOCTYPE, "re"))

Education Collection via ProQuest (added in 30 last days)
MAINSUBJECT.EXACT.EXPLODE("Meta Analysis") OR ab(meta-analysis OR systematic review) OR ti(meta-analysis OR systematic review)
